# Supplementary material for: Solution of Cross-Kerr Interaction Combined with Parametric Amplification
Source: Sci Rep. 2019 Feb 12;9:1830. doi: 10.1038/s41598-018-38377-7 (PMC6372670; doi:10.1038/s41598-018-38377-7)
Supplement: Supplementary file 1 — Supplementary Information [file 41598_2018_38377_MOESM1_ESM.pdf]

Supplementary Information: Theoretical Methods

Solution of Cross-Kerr Interaction Combined with Parametric Amplification

Sina Khorasani

S1 Construction of Noise Terms

It is straightforward to see how the noise terms of higher-order operators should be constructed. In the case that the operators are treated fully nonlinearly regardless of their mean values, then the corresponding noise spectral densities could be non-trivial to calculate. This issue, for the case of the squared operator $\hat{d}$ has been discussed in details elsewhere [S1]. But this is appropriate only when the noise spectral density is under consideration. When other quantities are to be measured, for which the non-squared ladder operators might be needed, an iterative approach like $\hat{b}_{j+1} = \hat{d} + \hat{b}_j - \frac{1}{2}\hat{b}_j^2$ with $\hat{b}_0 = \hat{1}$ provides uniform convergence to the ladder operator $\hat{b}$.

In order to do this, one may use, for instance, the Langevin equation for $\hat{b}$ and multiply both sides by $\hat{b}$ from left and right. Summing up together, leads to a Langevin equation for $\hat{b}^2$ with a noise input term such as those displayed in (10). The Langevin equation for $\hat{d}$ can be also directly constructed as shown in the article, and that ends up in a noise term as $\hat{d}_{\text{in}}$ with a decay rate of Γ. Within the accuracy of Langevin equations, these two noise terms coming from the two approaches should be identical, and this is how one can obtain all the noise terms in (10) in such an iterative manner alike.

One should keep in mind that the Langevin equations are neither exact nor rigorous by nature, as their construction necessitates at least two approximations of non-dispersive coupling and Gaussian white noise. The discussion around this topic is outside of the scope of the present study.

S2 Silence of Multiplicative Operators

The first issue to notice in treatment of the multiplicative noise is the dependence of the multiplying operators to the noise terms. These operators also are determined from lower order Langevin equations in which similar noise terms are fed in. Iteratively going back to the lowest order determines that these multiplying operators appear as an infinite series such as

$$\hat{a}(t) = \hat{a}_0(t) + a(t) + a_1(t)\hat{a}_{\text{in}}(t) + a_2(t)\hat{a}_{\text{in}}^2(t) + \dots, \tag{S1}$$

where $\hat{a}_0(t) = \exp\left[(i[\mathbf{M}] - [\Gamma])t\right]\hat{a}_0(0)$ is the decaying operator term of the homogeneous solution to the system of Langevin equations, which decays to zero, thus taking no part in the steady state solution, and is excluded from contributing to the noise spectral density. Furthermore, $a(t) = a(t)\hat{1}$ is the noiseless or silent part of the operator $\hat{a}(t)$ driven by the external classical pump field, which can in principle be determined from solving the Langevin equations with the stochastic terms dropped, while only keeping the drive terms as input.

The above term ultimately gets multiplied to another Gaussian white noise term, such as $\hat{a}_{\text{in}}(t)$ again. Such a multiplicative noise term as $\hat{a}(t)\hat{a}_{\text{in}}(t)$ will have an expansion given by

$$\hat{a}(t)\hat{a}_{\text{in}}(t) = a(t)\hat{a}_{\text{in}}(t) + a_1(t)\hat{a}_{\text{in}}^2(t) + a_2(t)\hat{a}_{\text{in}}^3(t) + \dots, \tag{S2}$$

and so on. It is not difficult to see that as long as a lower-order Gaussian noise term is present, the higher-order terms will have negligible contribution to non-zero absolute (and not detuned) frequencies in the ultimate noise spectral density. This is discussed in §S5.

In order to establish this, we may define the second-order noise corresponding to the squared process $\hat{c}(t) = \frac{1}{2}\hat{a}^2(t)$, which clearly has a decay rate of 2κ. The corresponding stochastic noise process is

$$\hat{c}_{\text{in}}(t) = \frac{1}{\sqrt{2\kappa}}\hat{a}_{\text{in}}^2(t). \tag{S3}$$

The stochastic process $\hat{c}_{\text{in}}(t)$ is no longer Gaussian white although $\hat{a}_{\text{in}}(t)$ is a Gaussian white stochastic process by assumption with a symmetrized auto-correlation $\langle \hat{a}^\dagger(t)\hat{a}(\tau)\rangle_{\text{S}} = \frac{1}{2}\delta(t-\tau)$. The symmetrized autocorrelation of this higher-order stochastic process in light of the Isserlis-Wick theorem [S1] is thus given by

$$\begin{aligned}\langle \hat{c}_{\text{in}}^\dagger(t)\hat{c}_{\text{in}}(\tau)\rangle_{\text{S}} &= \frac{1}{2\kappa}\langle \hat{a}_{\text{in}}^\dagger(t)\hat{a}_{\text{in}}^\dagger(\tau)\rangle_{\text{S}}\langle \hat{a}_{\text{in}}(t)\hat{a}_{\text{in}}(\tau)\rangle_{\text{S}} + 2\times\frac{1}{2\kappa}\langle \hat{a}_{\text{in}}^\dagger(t)\hat{a}_{\text{in}}(\tau)\rangle_{\text{S}}\langle \hat{a}_{\text{in}}^\dagger(t)\hat{a}_{\text{in}}(\tau)\rangle_{\text{S}} \\ &= \frac{1}{\kappa}\langle \hat{a}_{\text{in}}^\dagger(t)\hat{a}_{\text{in}}(\tau)\rangle_{\text{S}}^2 = \frac{1}{4\kappa}\delta^2(t-\tau).\end{aligned} \tag{S4}$$

The corresponding spectral density of this noise process, being its Fourier transform, simply causes a Dirac delta at zero frequency [S1]. Similarly, all higher-power noise processes will have no contribution to the non-zero frequency of the noise spectral density. As a result, the multiplicative noise (S2) can be effectively truncated as

$$\hat{a}(t)\hat{a}_{\text{in}}(t) = a(t)\hat{a}_{\text{in}}(t), \tag{S5}$$

without causing any error in the non-zero frequencies of the resulting noise spectral density. A more general treatment of the second-order noise processes with Gaussian resonances is discussed elsewhere [S1].

S3 Noise Spectral Density

Following the general approach to construction of the scattering matrix based on the input-output formalism [S1–S3], one may easily show that

$$\{\hat{A}_{\text{out}}(w)\} = \mathbf{S}(w)\{\hat{A}_{\text{in}}(w)\}, \tag{S6}$$

where $\mathbf{S}(w)$ is the 6×6 scattering matrix given by

$$\mathbf{S}(w) = \mathbf{I} - [\sqrt{\Gamma}]\,(i[\mathbf{M}] - [\Gamma] - iw\mathbf{I})^{-1}\,[\sqrt{\Gamma}]. \tag{S7}$$

Expansion of the output operator array gives

$$\hat{A}_{\text{out},j}(w) = \sum_{l=1}^{6} S_{jl}(w)\hat{A}_{\text{in},l}(w), \tag{S8}$$

where $\hat{A}_{\text{in},l}(w)$ are multiplicative noise terms such as $a_l(w)\hat{a}_{\text{in},l}(w)$, where $\hat{a}_{\text{in},l}$ stand for white Guassian White stochastic processes $\hat{a}_{\text{in}}$, $\hat{b}_{\text{in}}$, and their conjugates $\hat{b}^{\dagger}_{\text{in}}$, $\hat{b}^{\dagger}_{\text{in}}$, and also $a_l(w)$ are the corresponding Fourier-transformed silent multiplicative terms. This can be correspondingly shown to lead to the noise spectral densities

$$S_{AA,j}(w) = \sum_{l=1}^{6} \left|\frac{1}{\gamma_l} S_{jl}(w) * a_l(w)\right|^2 S_{A_lA_l}(w), \tag{S9}$$

with the understanding that the terms corresponding to conjugate noise operators are grouped together under the absolute value. Here, $S_{A_lA_l}(w)$ are the symmetrized noise spectral densities of the Gaussian White processes $\hat{a}_{\text{in},l}$. The spectral densities of these processes are typically constants as $\bar{n}_l + \frac{1}{2}$ with $\bar{n}_l$ being thermal occupation number of bosons. For an optical bosonic bath, one may conveniently set $\bar{n}_l = 0$, while for phonons $\bar{n}_l$ can be estimated from Bose-Einstein distribution [S4]. Furthermore, the symbol $*$ represents convolution in the frequency domain.

The approach provided here, leads to the noise spectral densities of higher-order operators $\hat{d} = \frac{1}{2}\hat{b}^2$, $\hat{d}^{\dagger} = \frac{1}{2}\hat{b}^{\dagger 2}$ and $\hat{m}$. Neither of these is the directly measurable spectrum, but it is rather the noise spectral density of ladder operator $\hat{b}$ for photons, which can be measured. These necessitates a way to recover the information through what is calculable by the method of higher-order operators.

The symmetrized noise spectral density of $\hat{d} = \frac{1}{2}\hat{b}^2$ is by definition given in terms of the Fourier transform of the corresponding symmetrized auto-correlation function, which is

$$\begin{aligned} S_{DD}(w) &= \frac{1}{2\pi}\int_{-\infty}^{+\infty} e^{iwt}\,\langle d^{\dagger}(\tau)d^{\dagger}(t+\tau)\rangle_{\text{S}}\,dw = \frac{1}{8\pi}\int_{-\infty}^{+\infty} e^{iwt}\,\langle b^{\dagger}(\tau)b^{\dagger}(\tau)b^{\dagger}(t+\tau)b^{\dagger}(t+\tau)\rangle_{\text{S}}\,dw \\ &= \frac{1}{4\pi}\int_{-\infty}^{+\infty} e^{iwt}\,\langle b^{\dagger}(\tau)b^{\dagger}(t+\tau)\rangle_{\text{S}}^2\,dw. \end{aligned} \tag{S10}$$

where the last expression is found by application of the Isserlis-Wick theorem and $\langle b^{\dagger}(\tau)b^{\dagger}(\tau)\rangle_{\text{S}} = 0$. By noting the definition of Fourier and inverse Fourier transforms, we get

$$S_{BB}(w) = \frac{1}{2} + \mathcal{F}\left\{\sqrt{\frac{1}{2}\mathcal{F}^{-1}\left\{S_{DD}(w) - \frac{1}{2}\right\}(t)}\right\}(w), \tag{S11}$$

where $\frac{1}{2}$ is substrated and added to account for the half a quanta of white noise which is lost in the symmetrization, and if not removed will cause appearance of a non-physical Dirac delta under the square root. While $S_{DD}(w)$ is found from simple scattering matrix calculations, all it takes now to find the measurable quantity $S_{BB}(w)$ is to take an inverse Fourier transform, followed by a square root and another Fourier transform. Similarly, one we may now define $\bar{S}_{BB}(w) = \frac{1}{2}[S_{BB}(w) + S_{BB}(-w)]$ as the symmetrized noise spectrum. The equation (S11) is the main key to recover the expected results from the higher-order operator algebra.

S4 Time-evolution of Operators

It is easy to obtain the explicit solution to the truncated system of Langevin equations (5)

$$\{A(t)\} = e^{i[\mathbf{N}]2\Omega t}\{A(0)\} - 2\Omega e^{i[\mathbf{N}]2\Omega t}\int_0^t e^{-i[\mathbf{N}]2\Omega\tau}\left(i\frac{\alpha}{2}\{A_c\} - \left[\sqrt{\Gamma}\right]\{A_{\text{in}}(\tau)\}\right)d\tau, \tag{S12}$$

where $\exp(\cdot)$ represents the matrix exponentiation, and $[\mathbf{N}] = [\mathbf{M}] + i[\Gamma]$.

S5 Non-negative Integer Powers of Noise

It is straightforward to see that any term involving a non-negative integer power of a noise such as $\hat{\alpha}_{\text{in}}^j(t); j \in \mathbb{N}, j > 1$ where $\hat{\alpha}_{\text{in}}^j(t) = \kappa^{\frac{1-j}{2}}\hat{a}_{\text{in}}^j(t)$ has identically zero contribution to the measured noise spectral density. In order to show this, let us the noise assume the normal autocorrelation

$$\langle\hat{\alpha}_{\text{in}}^{\dagger j}(\tau)\hat{\alpha}_{\text{in}}^j(t)\rangle_{\text{S}} = \zeta\exp\left[-\pi\zeta^2(t-\tau)^2\right]. \tag{S13}$$

In the limit of $\zeta \to +\infty$ this will settle back to the expected Dirac delta's function $\delta(t-\tau)$. The autocorrelation of the measurable optical field is connected to the operator $\hat{a}(t)$, which by means of the Isserlis-Wick theorem becomes

$$\langle\hat{a}^\dagger(\tau)\hat{a}(t)\rangle_{\text{S}} = \left[\frac{\kappa^{j-1}}{j}\langle\hat{a}_{\text{in}}^{\dagger j}(\tau)\hat{a}_{\text{in}}^j(t)\rangle_{\text{S}}\right]^{\frac{1}{j}} = \left(\frac{\kappa^{j-1}\zeta}{j}\right)^{\frac{1}{j}}\exp\left[-\frac{\pi\zeta^2}{j}(t-\tau)^2\right]. \tag{S14}$$

The corresponding noise spectral density in frequency domain, where w is the absolute optical frequency (and not the detuning referenced to a certain non-zero resonance frequency), is given by

$$\mathcal{F}\left\{\langle\hat{a}^\dagger(\tau)\hat{a}(t)\rangle_{\text{S}}\right\}(w) = \left(\frac{\kappa^{j-1}\zeta}{j}\right)^{\frac{1}{j}}\mathcal{F}\left\{\exp\left[-\frac{\pi\zeta^2}{j}(t-\tau)^2\right]\right\}(w)|_{\tau=0} = \frac{\sqrt{j}}{j^{\frac{1}{j}}}\left(\frac{\kappa}{\zeta}\right)^{\frac{j-1}{j}}\exp\left[-\frac{jw^2}{4\pi\zeta^2}\right], \tag{S15}$$

In the limit of $\zeta \to +\infty$ with $j > 1$ the above expression is identically zero, and hence meeting the claim.

It is equally straightbackward to show that for any white Gaussian noise such as $\hat{a}_{\text{in}}$ satisfying $\langle\hat{a}_{\text{in}}^\dagger(t)\hat{a}_{\text{in}}(\tau)\rangle_{\text{S}} = \frac{1}{2}\delta(t-\tau)$, the higher-power noise processes $\hat{\alpha}_{\text{in}}^j(t) = \kappa^{\frac{1-j}{2}}\hat{a}_{\text{in}}^j(t)$ contribute only to the zero frequency of the noise spectral density. To show this, we assume

$$\langle\hat{a}_{\text{in}}^\dagger(\tau)\hat{a}_{\text{in}}(t)\rangle_{\text{S}} = \frac{\zeta}{2}\exp\left[-\pi\zeta^2(t-\tau)^2\right], \tag{S16}$$

which again in the limit of $\zeta \to +\infty$ reproduces the Dirac's delta $\delta(t-\tau)$. Then the Isserlis-Wick theorem for such a Gaussian noise process could be exactly used to write

$$\langle\hat{\alpha}_{\text{in}}^{\dagger j}(\tau)\hat{\alpha}_{\text{in}}^j(t)\rangle_{\text{S}} = \frac{j}{2^j\kappa^{j-1}}\langle\hat{a}_{\text{in}}^\dagger(\tau)\hat{a}_{\text{in}}(t)\rangle_{\text{S}}^j = \frac{j\zeta^j}{2^j\kappa^{j-1}}\exp\left[-\pi j\zeta^2(t-\tau)^2\right]. \tag{S17}$$

Taking the Fourier transform from both sides gives the resulting noise spectral density

$$\mathcal{F}\left\{\langle\hat{\alpha}_{\text{in}}^{\dagger j}(\tau)\hat{\alpha}_{\text{in}}^j(t)\rangle_{\text{S}}\right\}(w)|_{\tau=0} = \frac{\sqrt{j}\zeta^{j-1}}{2^j\kappa^{j-1}}\exp\left[-\frac{w^2}{4\pi j\zeta^2}\right], \tag{S18}$$

which in the limit of $\kappa = \zeta \to +\infty$ yields an upper bound to a constant number of quanta $\sqrt{j}/2^j$, being less than $\frac{1}{2}$ for $j > 1$. This maximum bound to the background number of added noise quanta due to higher-power noise rapidly decays to zero with increasing j.

These limits are physically meaningful as long as cavity linewidth is much larger than the pump laser linewidth, which is quite accurately met in practice. So, when no squeezing is taking place and cavity resonances exhibit noise spectra corresponding to a much larger number of quanta than $\frac{1}{2}$, it should be safe to ignore the effect of square noise terms and higher powers.

Ultimately, a numerical integration carried out on a nonlinear differential equation with exaggerated noise input amplitude could very visibly distinguish the zero contribution of the higher-power noise terms, quite expectedly, confirming the general above conclusions.

S6 Multiplicative Noise

It is possible to make simple estimates for $b(w)$ to be used instead of $\bar{b}$ in (30), where a convolution such as (S9) would have been needed instead. This can be done by setting up the Langevin equations for operators $\{\hat{b},\hat{b}^\dagger\}$, which are coupled. Then these have to be Fourier-transformed and diagonalized to find $\hat{b}(w)$ explicitly, and the expectation of this expression $b(w)=\langle\hat{b}(w)\rangle$ can be now used as [S5]

$$S_{DD}(w)=\frac{1}{\gamma^2}|b(w)*[S_{11}(w)+S_{13}(w)]+b^*(w)*[S_{12}(w)+S_{13}(w)]|^2S_{YY}(w). \tag{S19}$$

This process is a lot more complicated and typically can be simplified by direct utilization of (30). Nevertheless, the corresponding Langevin equations are

$$\frac{d}{d\tau}\left\{\begin{array}{c}\hat{b}\\ \hat{b}^\dagger\end{array}\right\}=\frac{1}{2}\left[\begin{array}{cc}-i(2\beta\hat{n}+1)-\frac{1}{2}\gamma & -i4\alpha\\ i4\alpha & i(2\beta\hat{n}+1)-\frac{1}{2}\gamma\end{array}\right]\left\{\begin{array}{c}\hat{b}\\ \hat{b}^\dagger\end{array}\right\}-\sqrt{\gamma}\left\{\begin{array}{c}\sqrt{\gamma}\bar{b}+\hat{y}_{\text{in}}\\ \sqrt{\gamma}\bar{b}^*+\hat{y}_{\text{in}}^\dagger\end{array}\right\}. \tag{S20}$$

Replacing $\hat{n}$ with $\bar{n}$, taking the expectation and some simplification gives

$$\left\{\begin{array}{c}b(w)\\ b^*(w)\end{array}\right\}=\gamma([\mathbf{W}]-iw[\mathbf{I}])^{-1}\left\{\begin{array}{c}\bar{b}\\ \bar{b}^*\end{array}\right\}, \tag{S21}$$
$$[\mathbf{W}]=\frac{1}{2}\left[\begin{array}{cc}-i(2\beta\bar{n}+1)-\frac{1}{2}\gamma & -i4\alpha\\ i4\alpha & i(2\beta\bar{n}+1)-\frac{1}{2}\gamma\end{array}\right].$$

Here, the factor $\frac{1}{2}$ is included to take care of normalization with respect to 2Ω rather than Ω. Functions $b(w)$ and $b^*(w)$ from solution of (S21) can be plugged in the convolutions of (S19). Note the cancellation of γ as it explicitly appears in (S19) and (S21).

References

[S1] Khorasani, S. Higher-order interactions in quantum optomechanics: Analytical solution of nonlinearity. *Photonics* **4**, 48 (2017).
[S2] Gardiner, C.W. & Zoller, P. *Quantum Noise* (Springer, 2004).
[S3] Gardiner, C.W. & Collett, M.J. Input and output in damped quantum systems: Quantum stochastic differential equations and the master equation. *Phys. Rev. A* **31**, 3761 (1985).
[S4] Aspelmeyer, M., Kippenberg, T.J. & Marquardt, F. Cavity optomechanics. *Rev. Mod. Phys.* **86**, 1391 (2014).
[S5] Khorasani, S. Method of higher-order operators for quantum optomechanics. *Sci. Rep.* **8**, 11566 (2018).
